# MicroRNA-5112 Targets IKKγ to Dampen the Inflammatory Response and Improve Clinical Symptoms in Both Bacterial Infection and DSS-Induced Colitis

**DOI:** 10.3389/fimmu.2022.779770

**Published:** 2022-02-10

**Authors:** Xilong Kang, Yang Jiao, Yingying Zhou, Chuang Meng, Xiaohui Zhou, Li Song, Xinan Jiao, Zhiming Pan

**Affiliations:** ^1^ Jiangsu Key Laboratory of Zoonosis, Yangzhou University, Yangzhou, China; ^2^ Jiangsu Co-Innovation Center for the Prevention and Control of Important Animal Infectious Diseases and Zoonoses, Yangzhou University, Yangzhou, China; ^3^ Key Laboratory of Prevention and Control of Biological Hazard Factors (Animal Origin) for Agrifood Safety and Quality, Ministry of Agriculture of China (MOA), Yangzhou University, Yangzhou, China; ^4^ Joint International Research Laboratory of Agriculture and Agri-Product Safety of the Ministry of Education, Yangzhou University, Yangzhou, China; ^5^ Pathobiology and Veterinary Science, College of Agriculture, Health and Natural Resources, University of Connecticut, Storrs, CT, United States

**Keywords:** miR-5112, IKK-γ, inflammation, flagellin, *Salmonella*, DSS-induced colitis

## Abstract

Inflammation is a double-edged sword that can be induced by various PAMPs, resulting in the control of infection by invading pathogens or injuries. The inflammatory response requires strict and precise control and regulation. MicroRNAs (miRNAs) are small non-coding RNA molecules that regulate gene expression *via* translational inhibition or mRNA degradation. However, the role of miRNAs in inflammation induced by flagellin (ligand of TLR5) has yet to be fully determined. In this study, we identified differentially expressed miRNAs in murine bone marrow-derived dendritic cells (BMDCs) between flagellin treatment and medium alone using miRNA microarray. We found that flagellin stimulation downregulated miR-5112 expression in BMDCs and spleen DCs *in vitro* and *in vivo*. The overexpression of miR-5112 decreased inflammatory cytokine production, accompanied by a reduction of IKKγ in flagellin-stimulated BMDCs. We demonstrated that miR-5112 could directly target IKKγ to inhibit inflammatory cytokine production. Furthermore, miR-5112 inhibited the inflammatory response induced by flagellin or *Salmonella* infection *in vivo*. Interestingly, miR-5112 could also dampen the inflammatory response and alleviate dextran sulfate sodium (DSS)-induced colitis in C57BL/6 mice. These results suggest that miR-5112 could be a novel therapeutic target for both bacterial infection and DSS-induced colitis model.

## Introduction

Inflammation plays an important role in the control of pathogens, and it can be triggered by the recognition of pathogen-associated molecular patterns (PAMPs) *via* pattern recognition receptors (PRRs). A family of Toll-like receptor (TLR) members of PRRs act as primary sensors that detect a wide variety of microbial components and elicit inflammatory responses (1). TLR5, expressed on various immune cells, including macrophages, dendritic cells (DCs), can specifically recognize flagellin, which is the major structural protein of bacterial flagella. Upon activation, TLR5 triggers the MyD88-dependent signaling pathway and activates nuclear factor κB (NF-κB), leading to the production of proinflammatory cytokines or chemokines to defend against invading bacteria (2, 3). Although inflammation can defend against invading bacteria, it is also a double-edged sword. Deregulated or excessive inflammation can cause tissue injury, and inflammatory diseases (4). Therefore, there is a need for inflammation induced by TLR signaling pathways to be tightly regulated and precisely controlled.

MicroRNAs (miRNAs) are a class of small (approximately 18-25 nucleotides in length) and highly conserved noncoding RNAs. They can post-transcriptionally regulate gene expression by binding to 3’-untranslated regions (UTRs), resulting in translational repression or mRNA degradation (5). miRNAs have been confirmed to play key roles in many biological processes, including cell proliferation, differentiation, development, apoptosis, cancer, and tumorigenesis (6). In recent years, evidence has increasingly shown that miRNAs are involved in the regulation of inflammatory responses induced by the recognition of PAMPs *via* TLRs (7). For example, miR-155 can negatively regulate the TLR4-induced inflammatory response by targeting MYD88, TAB2, and IKKϵ, among others (8). miRNAs are thought to regulate TLR signaling at different levels by targeting multiple molecules involved in the TLR pathway, such as the expression of TLRs, TLR signaling molecules, TLR-induced transcription factors, regulators of the TLR signaling pathway and even the final functional cytokines of TLR signaling (9). Although the contributions of a few individual miRNAs to the TLR signaling pathway have been reported, the roles of miRNAs in precise fine-tuning of this process have yet to be fully elucidated. In particular, the regulatory role of miRNAs in flagellin (TLR5 ligand) induced inflammation has scarcely been reported and requires an in-depth investigation.

In the present study, we found that flagellin stimulation downregulated miR-5112 expression in murine BMDCs and spleen DCs (both *in vitro* and *in vivo*). miR-5112 negatively regulates flagellin-induced inflammatory cytokine production by targeting IKKγ in the TLR5 signalling pathway. Thus, we identified a potential positive feedback loop in which the flagellin-mediated miR-5112 reduction functioned to regulate DC activation characterized by enhanced inflammatory cytokine production. In addition, we found that administration of exogenous miR-5112 agomiR reduced *Salmonella*-induced inflammation and alleviated dextran sulfate sodium (DSS)-induced colitis in mice.

## Materials and Methods

### Mice and Ethics Statement

Six-week-old, specific-pathogen-free female or male C3H/HeJ and C57BL/6 mice were purchased from SLAC Laboratory Animal Co., Ltd. (Shanghai, China). The mice were housed in isolators, fed a pathogen-free diet and water, and exposed to a 12-hour light/dark cycles at a temperature of 23 ± 1°C. All animal experiments were approved by the Animal Welfare and Ethics Committees of Yangzhou University, and complied with the guidelines of the Institutional Administrative Committee and Ethics Committee of Laboratory Animals (SYXK [Su] 2017–0044).

### Generation and Culture of Bone Marrow-Derived Dendritic Cells

Murine bone marrow-derived dendritic cells (BMDCs) were generated from bone marrow progenitors as described previously (10, 11), with minor modifications. Briefly, bone marrow cells were extracted from the tibial and femoral bones of C3H/HeJ mice, and red blood cells were removed using ACK Lysing Buffer (Life Technologies, Carlsbad, CA, USA). The cells were cultured at a density of 1 × 10^6^ cells/ml in RPMI 1640 medium supplemented with 10% fetal bovine serum, 100 μg/ml streptomycin, 100 U/ml penicillin, 50 μM mercaptoethanol (Life Technologies), 10 ng/ml recombinant mouse granulocyte-monocyte colony-stimulating factor (rmGM-CSF) and 1 ng/ml recombinant mouse IL-4 (R&D Systems, Minneapolis, MN, USA) in tissue culture dishes. Half of the medium was replaced on days 3 and 5. On day 7, non-adherent and loosely adherent cells were harvested, and the CD11c^+^ BMDCs were sorted using anti-CD11c-coated magnetic beads with the auto-MACS system (Miltenyi Biotec, Bergisch Gladbach, Germany). The purity of the sorted CD11c^+^ BMDCs was confirmed by CD11c staining (anti-CD11c-phycoerythrin (PE); BD Biosciences, San Diego, CA, USA) and detection by flow cytometry (FCM). The sorted CD11c^+^ BMDCs were used as immature BMDCs (imBMDCs) in the experiments. The cells were then cultured in the presence of flagellin from *Salmonella typhimurium* (endotoxin level < 0.05 EU/μg; *In vivo*Gen, San Diego, CA, USA) at a final concentration of 100 ng/ml or in medium alone for 24 h. All generations and cultures of cells were incubated at 37°C in a 5% CO_2_ atmosphere.

### Cytokine Analysis by ELISA

The culture supernatants of BMDCs after 24 h of treatment with flagellin or medium alone were harvested and stored -70°C until use. The IL-12 p40 levels in the culture supernatant were determined by ELISA using a mouse IL-12 p40 ELISA Kit (BD Biosciences) according to the manufacturer’s instructions.

### Analysis of Cell Surface Molecule Expression by FCM

The surface molecule expression of BMDCs after 24 h of treatment with flagellin or medium alone was investigated by FCM as described previously (12). Briefly, the cells were harvested and stained with PE-labelled anti-CD11c and one of the following biotin-labelled antibodies for 20 min: anti-CD40, anti-CD80, or anti-CD86 (BD Biosciences). After washing twice, the cells were incubated with APC-conjugated streptavidin for 20 min. All incubations and washes were performed in PBS containing 0.5% BSA at 4°C. Finally, the cells were washed and resuspended in PBS containing 0.5% BSA for determination of surface molecular expression using a FACSCalibur flow cytometer (BD Biosciences). FCM data were analyzed using FlowJo software 7.6 (Tree Star, San Carlos, CA, USA).

### miRNA Microarray Analysis

Total RNA, including miRNA, was purified from BMDCs after 24 h of treatment with flagellin or medium alone using the mirVana RNA isolation kit (Ambion, Austin, TX, USA) and used for analysis of miRNA expression profiling. miRNA array profiling was performed by CapitalBio Corp. (Beijing, China) using the Mouse miRNA Microarray, Release 18.0, 8 × 60 K (Agilent Technologies, Palo Alto, CA, USA). The purified RNA was labelled with Cy3, and hybridization was performed according to the manufacturer’s instructions. After washing, the arrays were scanned using an Agilent Scanner G2565CA (Agilent Technologies). The total gene signals were extracted using Agilent Feature Extraction software 10.7 (Agilent Technologies) and further analyzed with GeneSpring software (version 11.0; Agilent Technologies). The miRNA microarray data was deposited in the Gene Expression Omnibus (GEO) database (accession number GSE188656). A log transformation was performed with base 2. Student’s *t-*test was used to evaluate flagellin-stimulated cells relative to the medium control. Differentially expressed miRNAs were then identified based on the fold change and *P-*values calculated using the *t*-test. The miRNAs were defined as significantly up- or down-regulated if the *t*-test *P*-values were less than 0.05, and the fold changes were greater than 2-fold. Target genes of differentially expressed miRNAs were predicted using TargetScan 6.2 (http://www.targetscan.org/mmu_61/).

### Quantitative Reverse Transcription and Real-Time PCR Analysis of miRNA

Quantitative real-time PCR (qRT-PCR) was used to validate miRNA expression changes, as previously described (13). Total RNA was extracted using TRIzol reagent (Invitrogen, Carlsbad, CA, USA) according to the manufacturer’s protocol. Next, reverse transcription reactions were performed with the PrimeScript RT Reagent Kit (Perfect Real Time, TaKaRa, Biotechnology Co. Ltd., Dalian, China) using miRNA-specific reverse transcription primers (**Table S1**). Quantitative PCR (qPCR) was performed on an ABI PRISM 7500 Real-Time PCR System (Applied Biosystems, Foster City, CA, USA) using SYBR Premix Ex Taq™ II (Perfect Real Time, TaKaRa). The qPCR primers for miR-5112, miR-193-5p, miR-466i-3p, and miR-3091-5p are listed in **Table S1**. The relative expression level of miRNAs was normalized to that of the internal control U6 small nuclear RNA using the 2^-^ *^ΔΔ^* ^Ct^ method (14).

### Detection of the Expression of miR-5112 in Spleen DCs

To analyze the expression of miR-5112 in spleen DCs (spDCs) *in vitro*, spDCs were isolated with anti-CD11c-coated magnetic beads using the auto-MACS system (Miltenyi Biotec), as previously described (15). Briefly, the spleens of C3H/HeJ mice were removed and perfused with 400 U/ml collagenase type IV (Invitrogen) containing 50 μg/ml DNase I (Invitrogen), and single spleen cell suspensions were prepared. The CD11c^+^ spDCs were sorted using anti-CD11c-coated magnetic beads according to the manufacturer’s instructions. Next, the spDCs were cultured in the presence of flagellin at a final concentration of 100 ng/ml or in medium alone for 24 h, and the cytokine IL-6 and IL-12 p40 levels in the culture supernatant were determined by ELISA, and the expression of miR-5112 *in vitro* was determined by qRT-PCR.

To analyze the expression of miR-5112 in spDCs *in vivo*, groups of three C3H/HeJ mice were intraperitoneally immunized with 2 μg of flagellin or PBS (used as a control). Twenty-four hours after immunization, spDCs were isolated as described above, andthe IL-6, IL-12 p40 mRNA levels and the expression of miR-5112 *in vivo* were determined by qRT-PCR.

### Dual Luciferase Reporter Assay

The wild-type murine IKKγ 3’-UTR luciferase reporter vectors harboring IKKγ sequences were constructed using cDNA from BMDCs. Two gene fragments (1081-1749, and 4321-5330) containing the partial 3’-UTR of murine IKKγ (GenBank ID: NM_010547.2) were amplified and cloned into the *Xba*I site of the pGl3-promoter vector (Promega, Madison, WI, USA), designated as pGL3-IKKγ 3’ UTR-WT-1 (containing miR-5112 target sites 1, 1336-1342), and pGL3-IKKγ 3’ UTR-WT-2 (containing miR-5112 target sites 2 (4657–4663), and 3 (4866-4873)), respectively. The predicted target sites were subjected to mutagenesis to inducea deletion (pGL3-IKKγ 3’ UTR-MU-1, and pGL3-IKKγ 3’ UTR-MU-2)for use as the control. HEK293T cells (Cell Bank of Type Culture Collection of Chinese Academy of Sciences, Shanghai, China) were co-transfected with 200 ng of luciferase reporter plasmid, 80 ng of pRL-TK *Renilla* luciferase plasmid, and the miR-5112 mimics or control mimics (final concentration, 20 nM; GenePharma, Shanghai, China). After 24 h, luciferase activity was measured using the Dual-Luciferase Reporter Assay System (Promega) according to the manufacturer’s instructions. Firefly luciferase activity was normalized to *Renilla* luciferase activity.

### Cell Transfection

miR-5112 mimics (dsRNA oligonucleotides) and miR-5112 inhibitors (single-stranded chemically modified oligonucleotides) from GenePharma were used for the overexpression and inhibition of miR-5112 activity in murine BMDCs, respectively. BMDCs were transfected with miR-5112 mimics or inhibitors at concentrations of 30 nM or 100 nM, respectively. Negative control mimics or inhibitors (GenePharma) were used as matched controls. Transfection was performed using Lipofectamine 2000 (Invitrogen), according to the manufacturer’s protocol. After 24 h, the cells were stimulated with flagellin at a final concentration of 100 ng/ml or with medium alone for another 24 h. The IL-12 p40 level in the culture supernatant was determined by ELISA using a mouse IL-12 p40 ELISA Kit (BD Biosciences) according to the manufacturer’s instructions.

### Western Blotting

Western blotting was used to assess IKKγ expression in BMDCs, as described previously (16). Lysates from transfected BMDCs following 24 h of treatment with flagellin or medium alone were denatured and subjected to SDS-PAGE. Proteins were electrotransferred onto a nitrocellulose membrane (Millipore, Bedford, MA, USA). The membrane was incubated with primary antibodies against IKKγ or β-actin (Abcam, Cambridge, UK), followed by hybridization with a secondary HRP-conjugated antibody. The blot signals were detected using Super Signal West Pico Chemiluminescent Substrate (Pierce, Rockford, IL, USA) according to the manufacturer’s instructions.

### miRNA Agomir or Antagomir Administration *In Vivo*

miRNA agomiRs and antagomiRs are chemically modified and cholesterol-conjugated stable miRNA mimics or inhibitors. AgomiR-5112 and antagomiR-5112 purchased from GenePharma were used for overexpression and inhibition of miR-5112 activity *in vivo*, respectively. AgomiR-5112 or antagomiR-5112 were delivered into C57BL/6 mice by intraperitoneal injection at a dose of 2.5 nmol or 25 nmol, respectively. PBS was used as a control. Twenty-four hours after injection, the mice were intraperitoneally immunized with 2 μg of flagellin. Two hours after immunization, individual mice were bled by retro-orbital plexus puncture. Serum samples were prepared by centrifugation and stored –20°C until analysis. The cytokines in serum were detected using the BD™ CBA Mouse Inflammation Kit and mouse IL-12 p40 ELISA Kit (BD Biosciences) according to the manufacturer’s instructions.

### *Salmonella* Challenge and Treatment

A schematic of the experimental design is provided in **Figure 6A**. Six-week-old female C57/BL6 mice (n=12) received 200 μl of PBS or PBS containing 2.5 nmol of agomiR-5112 *via* intraperitoneal injection on three consecutive days (day 1 to 3) (17). The mice were then infected with *Salmonella enterica* serovar *enteritidis* (*S*. *enteritidis*) strain C50336 obtained from the National Institute for the Control of Pharmaceutical and Biological Products (Beijing, China), as described previously (18). Briefly, the mice were treated with 7.5 mg of streptomycin by oral gavage on day 3. Twenty-four hours later, the mice were infected with 5 ×10^4^ CFU of C50336 (100-μl of bacterial suspension in PBS) or treated with 100 μl of sterile PBS (control) by oral gavage. The mice were monitored daily for survival and body weight loss. Three mice per group were sacrificed at 8 hours and 4 days post-infection. Serum was collected for inflammatory cytokine analysis using Millipore Milliplex MAP Mouse Cytokine/Chemokine Panel I (Millipore) according to the manufacturer’s protocol. Spleen, liver, ileum, cecum and colon were collected and used for the analysis of *Salmonella* loads and histopathological examination. The*Salmonella* loads in the organs were analyzed as described previously (19). Briefly, organ samples were aseptically collected, weighed, and homogenized in 1 ml of PBS, and serial 10-fold dilutions of tissue homogenates (100 μl of each) were plated on XLT4 agar (Difco Laboratories, Detroit, MI, USA) and incubated 37°C for 12-16 h. Bacteria were counted, and the numbers were expressed as log_10_ CFU/g. To analyze the histopathology (20), organ samples were fixed in 10% neutral-buffered formalin. After fixation, tissues were embedded in paraffin using conventional methods. Next, serial tissue sections (4 μm thick) were prepared and stained with hematoxylin and eosin (H&E) for histological analysis.

### Dextran Sulfate Sodium (DSS)-Induced Colitis and Treatment

DSS (MW 36000-50000, MP Biomedicals, Solon, OH, USA) was added to the drinking water (4%, w/v) and consistently given drinking for 6 days to induce colitis. 5-ASA was used as a positive control to treat the colitis. A schematic of the experimental design is shown in **Figure 7A**. Eight-week-old male C57BL/6 mice were randomly equally divided into five groups (n=5 per group): (1) control group, free to drinking water; (2) DSS group, 4% DSS in drinking water; (3) DSS+agomiR-5112 (2.5 nmol) group, 4% DSS in drinking water and treatment with 2.5 nmol agomiR-5112 by intraperitoneal injection quaque die; (4) DSS+agomiR-5112 (5.0 nmol) group, 4% DSS in drinking water and treatment with 5.0 nmol agomiR-5112 by intraperitoneal injection quaque die; (5) DSS+5-aminosalicylic acid (5-ASA; Sigma, St. Louis, Mo, USA) group, 4% DSS in drinking water and treatment with 5-ASA (100 mg/kg) by oral gavage quaque die. Body weight, stool constancy, and rectal bleeding were monitored daily. The severity of colitis was assessed daily using the disease activity index (DAI), which was calculated according to the following formula: DAI = (weight loss score + diarrhoea score + bleeding score)/3 (21). The following parameters were used to calculate the DAI (22): weight loss score (0, normal; 1, 0%-5%; 2, 5%-10%; 3, 10%-20%; 4, >20%), diarrhea score (0, normal; 2, loose stools; 4, watery diarrhea), and bleeding score (0, normal; 2, positive hemoccult; 4, gross bleeding). Serum was collected on days 3 and 6 by retro-orbital plexus puncture and used for cytokine determination with the BD™ CBA Mouse Inflammation Kit. On day 6, all mice were euthanized, and the entire colon was excised from the cecum to the anus. The colon length was measured. The partial colon tissue was fixed in 10% neutral-buffered formalin for histological analysis using H&E staining. To determine local inflammation in the colon, the concentrations of cytokines and MPO were determined using the BD™ CBA Mouse Inflammation Kit and Mouse MPO ELISA Kit (Sigma) according to the manufacturer’s instructions. Briefly, colons (100 mg wet weight) were homogenized in 500 μl of ice-cold PBS. The resulting homogenates were centrifuged 2000 × *g* for 20 min 4°C. The supernatants were harvested and used to determine the levels of cytokines and MPO.

### Statistical Analysis

All experiments were repeated, and the results were analyzed statistically using the Student’s *t*-test with Prism 5.0 software (GraphPad Inc., San Diego, CA, USA). Values are expressed as the mean± SEM, and significant differences were assigned to *P*-values < 0.05, < 0.01, and < 0.001 denoted by *, **, and ***, respectively.

## Results

### Generation and Sorting of Highly Pure CD11c^+^ BMDCs

Bone marrow cells were induced in the presence of rmGM-CSF and rmIL-4 for 7 days and showed a high percentage of CD11c^+^ cells (approximately 71.7%), indicating the successful generation of BMDCs (**Figure S1A**). To obtain highly pure CD11c^+^ BMDCs, the cells were further purified by sorting on an auto-MACS system using anti-CD11c-coated magnetic beads. More than 95% of the CD11c^+^ BMDCs were obtained after sorting, as assessed by flow cytometry (FCM) (**Figure S1B**). The sorted highly pure CD11c^+^ BMDCs were used as immature BMDCs in this study.

### Flagellin Induces Activation of BMDCs

Flagellin, a Toll-like receptor 5 (TLR5) agonist, can induce the production of proinflammatory cytokines and the upregulation of costimulatory molecules. To verify the activation of BMDCs, the immature BMDCs were incubated with 100 ng/ml of flagellin or with medium alone for 24 h. The expression of DC maturation markers, including the costimulatory molecules (CD80 and CD86) and the TNF-receptor family member CD40, was analyzed by FCM. As shown in **Figure S2A**, flagellin increased the expression of CD40. The mean fluorescence intensity (MFI) associated with CD40 significantly increased in proportion to the percentage of positive cells in BMDCs treated with flagellin (*P* < 0.05) (**Figure S2B**). At the same time, we measured inflammatory cytokine IL-12 p40 production in the culture supernatant of BMDCs following flagellin stimulation for 24 h. In the BMDC culture supernatant, flagellin stimulation significantly increased IL-12 p40 production compared to stimulation with medium alone (*P* < 0.001) (**Figure S2C**). Taken together, these data indicate that BMDCs are activated upon stimulation with flagellin.

### Flagellin-Activated BMDCs Downregulate miR-5112 Expression

**Figure 1:**
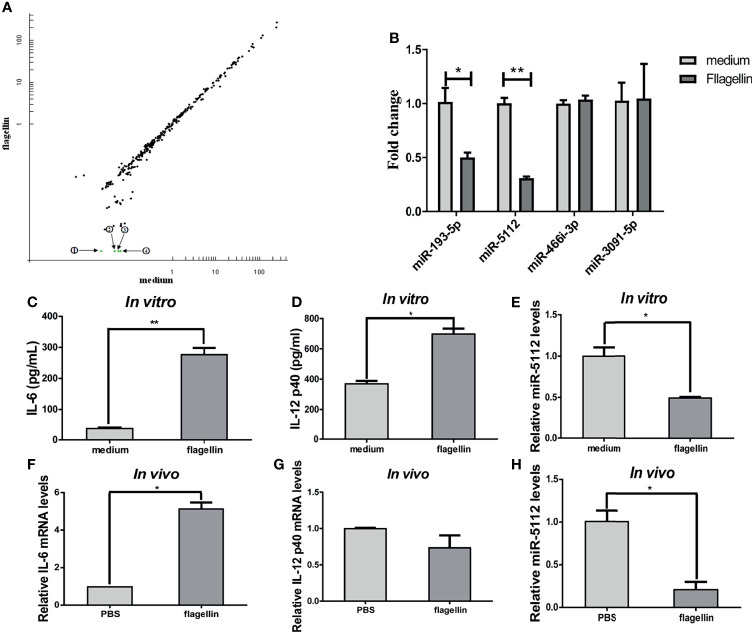
miRNA expression analysis in flagellin-activated DCs. **(A)** Microarray analysis of the miRNA expression profile in BMDCs treated with flagellin or with medium alone. The scatter plot shows the variation in miRNAs. Each plot represents one miRNA probe. The green dots represent a signal intensity ≤0.5 ratio between flagellin-treated and medium-treated BMDCs, indicating significantly downregulated miRNAs. ① miR-193-5p, ② miR-5112, ③ miR-466i-3p, and ④ miR-3091-5p. **(B)** Quantitative real-time PCR (qRT-PCR) to validate the miRNA expression profile in flagellin-treated BMDCs normalized to U6 small nuclear RNA levels. **(C–E)** Spleen DCs were isolated from three C3H/HeJ mice spleen using anti-CD11c-coated magnetic beads and stimulated with flagellin (100 ng/ml) for 24 h. The IL-6 **(C)** and IL-12 p40 **(D)** production in culture supernatants was measured by ELISA. The expression of miR-5112 was measured by qRT-PCR **(E)**. **(F–H)** Spleen DCs were isolated from C3H/HeJ mice (n=3/group) immunized with flagellin using anti-CD11c-coated magnetic beads, and the IL-6 **(F)**, IL-12 p40 **(G)** mRNA level and miR-5112 **(H)** was measured by qRT-PCR. The data shown represent the means ± SEM from 3 independent experiments. *P < 0.05, **P < 0.01.

miRNAs are short non-coding RNAs that bind to the 3’UTR of their target mRNAs and suppress target expression at the post-transcriptional level. We determined the expression profiles of miRNAs using microarrays in BMDCs treated with flagellin or with medium alone. As shown in **Figure 1A**, four differentially expressed miRNAs were identified by comparing the miRNA expression profiles of BMDCs treated with flagellin and medium alone. All four miRNAs (miR-193-5p, miR-466i-3p, miR-3091-5p, and miR-5112) were downregulated in BMDCs stimulated with flagellin for 24 h compared to those treated with medium alone (**Figure 1A**). We further validated the expression of the four miRNAs using qRT-PCR, and the results showed that the expression of miR-5112 and miR-193-5p was significantly downregulated in the flagellin-treated BMDCs (3.23- and 1.99-fold change, respectively) compared to the medium control (**Figure 1B**), which was consistent with the array data. However, qRT-PCR showed that the expression of miR-466i-3p and miR-3091-5p remained almost unchanged in flagellin-treated BMDCs compared with medium-treated BMDCs (**Figure 1B**), indicating that the microarray might have produced false-positive results and requires further validation by other assays. Considering that miR-5112 had the highest differential expression in flagellin-treated BMDCs, in this study, we focused on the characterization of miR-5112. We further detected the expression of miR-5112 in spleen DCs both *in vitro* and *in vivo*. The levels of IL-6 and IL-12 p40 were significantly increased in flagellin-treated spleen DCs compared with the control both *in vitro* (**Figures 1C, D**) and *in vivo* (**Figures 1F, G**). At the same time, miR-5112 expression was significantly decreased in flagellin-treated spleen DCs compared with the control, both *in vitro* (**Figure 1E**) and *in vivo* (**Figure 1H**).

### Enforced Overexpression of miR-5112 Inhibited the Flagellin-Induced Inflammatory Response in BMDCs

**Figure 2:**
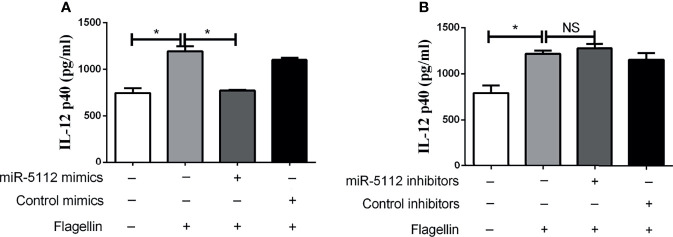
miR-5112 inhibited IL-12 p40 production in flagellin-stimulated BMDCs. BMDCs were transfected with miR-5112 mimics **(A)** or inhibitors **(B)** at a concentration of 30 or 100 nM, respectively. Negative control mimics or inhibitors were transfected as matched controls at the indicated concentrations. After 24 h, the cells were stimulated with flagellin. The production of IL-12 p40 in culture supernatants of BMDCs was measured by ELISA using a mouse IL-12 p40 ELISA Kit. The data shown represent the means ± SEM from 3 independent experiments. *P < 0.05; NS, not significant.

Given that miR-5112 was significantly downregulated in flagellin-activated BMDCs, we evaluated whether miR-5112 is involved in the regulation of BMDC activation. To this end, we overexpressed miR-5112 in murine BMDCs by transfecting the cells with miR-5112 mimics and then measured IL-12 p40 production (a hallmark of BMDC activation) in BMDCs followed by flagellin stimulation. Compared with the control mimics, miR-5112 mimics markedly decreased IL-12 p40 production in BMDCs stimulated with flagellin (**Figure 2A**). Simultaneously, IL-12 p40 production was measured in flagellin-stimulated BMDCs after transfection with miR-5112 inhibitors. However, miR-5112 inhibitors had no obvious effect on IL-12 p40 production in BMDCs stimulated with flagellin (**Figure 2B**).

### Exogenous miR-5112 AgomiR Inhibited the Flagellin-Induced Inflammatory Response *In Vivo*

**Figure 3:**
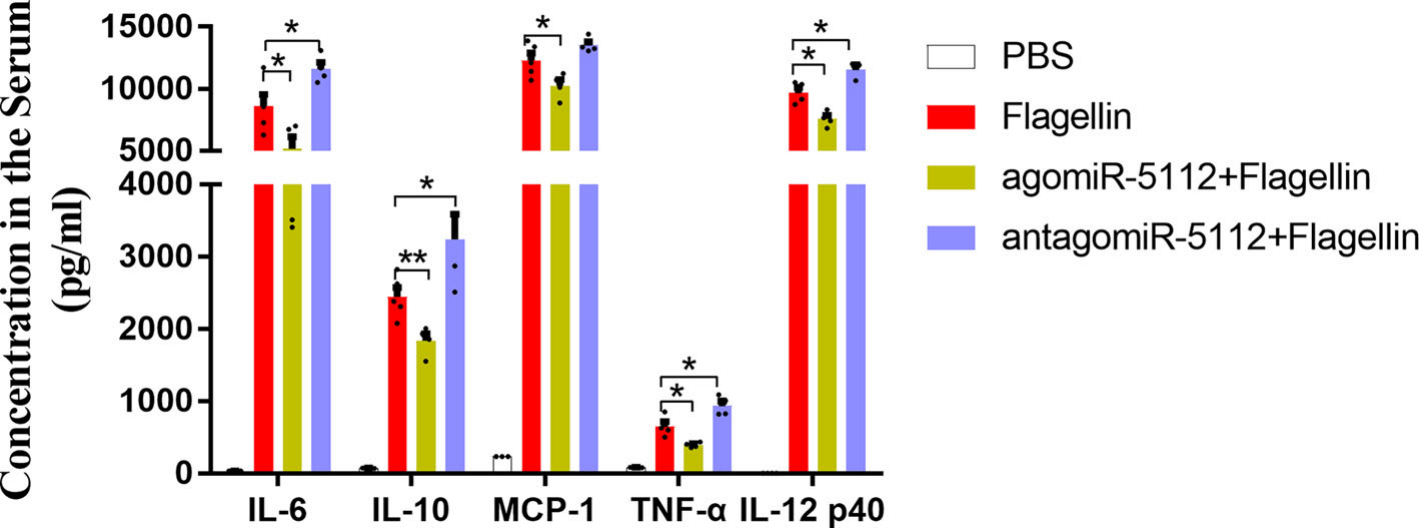
Exogenous miR-5112 agomiR inhibits the cytokine production induced by flagellin *in vivo*. C57BL/6 mice (n=5/group) were intraperitoneally injected with agomiR-5112 or antagomiR-5112 at doses of 2.5 nmol or 25 nmol, respectively. PBS was used as a control. After 24 h, the mice were immunized with 2 μg of flagellin. Serum samples were prepared following immunization for two hours. Serum cytokine levels were measured using the BD™ CBA Mouse Inflammation Kit and mouse IL-12 p40 ELISA Kit. The data are presented as the means ± SEM for each group. *P < 0.05; **P < 0.01.

To elucidate the function of miR-5112 *in vivo*, we intraperitoneally administered mice with miR-5112 agomiR or antagomiR to overexpress or inhibit miR-5112. Subsequently, the mice were treated with flagellin, and the cytokine levels were measured. Our data showed that the flagellin-induced inflammatory cytokines IL-6, IL-12 p40, and TNF-α were significantly decreased after administration with miR-5112 agomiR and increased after administration with miR-5112 antagomiR (**Figure 3**). The production of the chemokine MCP-1 induced by flagellin was also significantly decreased by miR-5112 agomiR. In addition, miR-5112 agomiR also decreased the production of the anti-inflammatory cytokine IL-10 induced by flagellin, while miR-5112 antagomiR enhanced IL-10 production (**Figure 3**).

### miR-5112 Targeted IKKγ in Flagellin-Stimulated BMDCs

**Figure 4:**
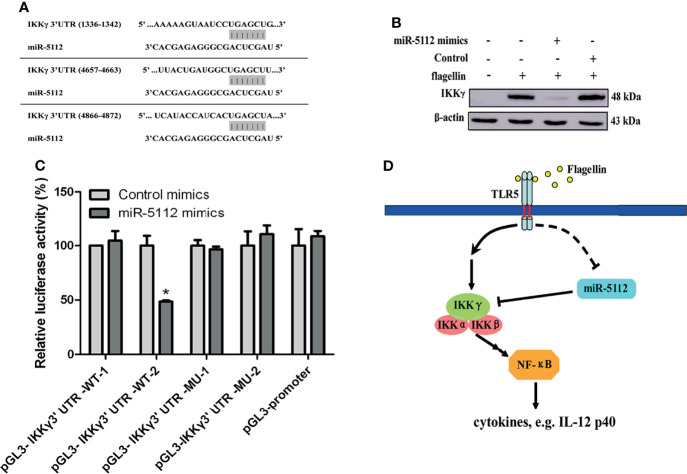
miR-5112 directly targets the IKKγ mRNA 3’ UTR. **(A)** Potential target sites of miR-5112 in IKKγ mRNA 3’ UTR are shown. The binding sites of miR-5112 were predicted by TargetScan. **(B)** BMDCs were transfected with miR-5112 mimics and control mimics (30 nM). After 24 h, the cells were stimulated with flagellin. The IKKγ protein expression was detected by Western blotting. β-actin served as a loading control. **(C)** HEK293T cells were cotransfected with wild-type IKKγ 3’UTR reporters (pGL3-IKKγ 3’ UTR-WT-1, 2) or the indicated mutants (pGL3-IKKγ 3’ UTR-MU-1, 2) and pTK-RL plasmid, together with miR-5112 mimics or control mimics. After 24 h, firefly luciferase activity was measured and normalized to *Renilla* luciferase activity. The data shown represent the means ± SEM from 3 independent experiments. *P < 0.05. **(D)** A proposed working model depicting a potential miR-5112 regulatory mechanism for inflammatory production in flagellin-activated DCs. The miR-5112 expression is downregulated in DCs after flagellin stimulation, which in turn promotes the production of inflammatory cytokines by targeting IKKγ. The reduction of miR-5112 is a potential positive feedback loop to enhance inflammatory cytokine production in flagellin-activated DCs.

Next, we examined the possible targets of miR-5112, that may modulate flagellin-induced cytokine production both *in vitro* and *in vivo*. Flagellin can induce the release of proinflammatory cytokines and chemokines through the recognition of TLR5 (23). Thus, we searched for the target gene of miR-5112 in the TLR5/NF-κB signalling pathway. As predicted by computational prediction using TargetScan (http://www.targetscan.org/mmu_61/), one of the potential targets of miR-5112 is the regulatory non-enzymatic scaffold protein NEMO (NF kappa B essential modulator), also known as IKKγ, which is an important intermediate recruited in canonical NF-κB signal transduction pathways. The computational prediction results revealed three putative miR-5112 target sites in the IKKγ 3’UTR (**Figure 4A**). The IKKγ protein expression level was significantly upregulated (**Figure 4B**), while the expression of miR-5112 was downregulated (**Figure 1B**) in flagellin-treated BMDCs and displayed a negative correlation between the expression of IKKγ and miR-5112, indicating that miR-5112 may negatively regulate IKKγ. This notion was supported by the finding that IKKγ expression was decreased by miR-5112 mimics (**Figure 4B**). Next, we evaluated whether miR-5112 could directly target IKKγ through 3’ UTR interactions using a dual luciferase reporter assay. First, we attempted to generate an miRNA reporter containing the full-length IKKγ 3’ UTR (≈5419 bps), but our attempts were unsuccessful. Instead, the pGL3-IKKγ 3’ UTR-WT-1 containing miR-5112 target sites 1 (1336-1342) and pGL3-IKKγ 3’ UTR-WT-2 containing miR-5112 target sites 2 (4657-4663) and 3 (4866-4873) were constructed. The results of the dual luciferase reporter assay showed that pGL3-IKKγ 3’ UTR-WT-2, but not pGL3-IKKγ 3’ UTR-WT-1, demonstrated a 51.5% reduction in luciferase reporter activity in the presence of miR-5112 mimics (**Figure 4C**). In addition, the deletion of putative target sites 2 and 3 in pGL3-IKKγ 3’ UTR-WT-2 abolished the reduction in luciferase reporter activity in the presence of miR-5112 mimics (**Figure 4C**). These data showed that miR-5112 could regulate the expression of IKKγ by directly targeting the 3’ UTR.

### Exogenous miR-5112 AgomiR Suppresses *Salmonella*-Induced Inflammation and Delays *Salmonella*-Induced Death in a Streptomycin Pre-Treated Mouse Model

**Figure 5:**
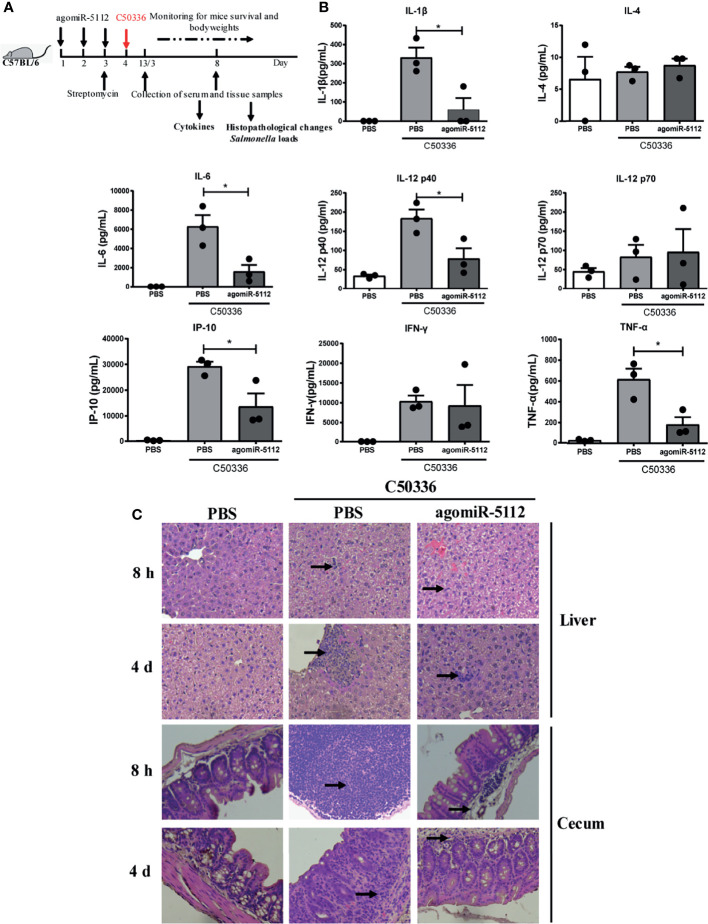
Exogenous miR-5112 agomiR inhibits the inflammatory response induced by *S*. *enteritidis* infection in mice. **(A)** Schematic diagram of the experimental design. AgomiR-5112 was delivered into C57/BL6 mice (n=12) by intraperitoneal injection on three consecutive days (day 1 to 3). Additionally, 7.5 mg of streptomycin was administered to the mice by oral gavage on day 3. After 24 h, the mice were infected with 5 ×10^4^ CFU of C50336 (100 -μl of bacterial suspension in PBS) or treated with 100 μl of sterile PBS (control) by oral gavage. At 8 hours and 4 days after *Salmonella* infection, three mice per group were sacrificed, and serum, liver, and cecum were collected. **(B)** The cytokines in serum from mice (n=3/group) at 4 days after *Salmonella* infection were measured using the Millipore Milliplex MAP Mouse Cytokine/Chemokine Panel I according to the manufacturer’s instruction. Data are expressed as the mean ± SEM. *P < 0.05. **(C)** Histopathological changes in liver and cecum of mice at 8 hours and 4 days after *Salmonella* infection examined by H&E staining and observed at ×100 magnification using an optical microscope. Arrows indicate inflammatory cell infiltration.

**Figure 6:**
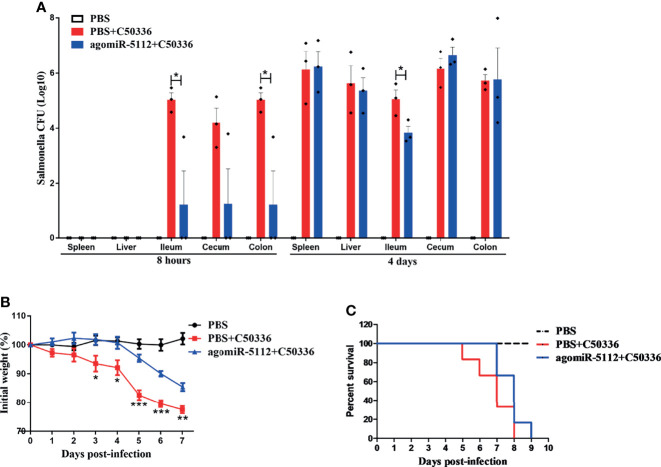
Exogenous miR-5112 agomiR delays mouse death induced by *S*. *enteritidis* infection. The agomiR-5112 injection and *Salmonella* infection procedures were performed as described in **Figure 6**. **(A)** *Salmonella* loads in mice organs (spleen, liver, ileum, cecum and colon, n=3/group) were determined at 8 h and 4 d after *Salmonella* infection. The number of bacteria was expressed as log_10_ CFU/g. Data are expressed as the mean ± SEM. *P < 0.05. **(B)** Changes in body weight of mice (n=6/group) were monitored daily for 7 days after *Salmonella* infection. Data are expressed as the mean ± SEM. **P < 0.01; ***P < 0.001 versus PBS+C50336 group **(C)** The survival of mice (n=6/group) was monitored daily after *Salmonella* infection.

*Salmonella* infections can cause diarrhea, inflammation, gastroenteritis, and systemic infections (24, 25). *Salmonella enterica* serovar *enteritidis* (*S*. *enteritidis*) is one of the most common serotypes of salmonellosis in humans. We analyzed the miR-5112 expression in peritoneal macrophages after the mice were infected with *S. enteritidis* for 8 h. The result showed that miR-5112 expression was decreased in macrophages of mice after *Salmonella* infection (**Figure S3**). We also evaluated the miR-5112 levels after the mice were intraperitoneal injected 2.5 nmol of agomiR-5112 *via* ion on three consecutive days. Compared with that of the PBS control group, the miR-5112 levels in peritoneal macrophages, spleen and colon of agomiR-5112 group increased 220.74-, 2.55- and 5.40-fold, respectively (**Figure S4**). Since the miR-5112 agomiR can reduce the flagellin-induced inflammatory response, we investigated whether the miR-5112 agomiR could also reduce *Salmonella*-induced inflammation using a streptomycin-treated mouse model. The streptomycin-treated mice received the miR-5112 agomiR and were then infected with *S*. *enteritidis* (**Figure 5A**). Cytokines including IL-1β, IL-6, IL-12 p40, IP-10, IFN-γ, and TNF-α were increased in *S*. *enteritidis*-infected mice at 4 days post-infection (**Figure 5B**). Importantly, the miR-5112 agomiR markedly inhibited the production of these cytokines, including IL-1β, IL-6, IL-12 p40, IP-10, and TNF-α, in *S*. *enteritidis*-infected mice (**Figure 5B**). Histopathological analysis showed that at 8 h and 4 days post-infection, the liver and cecum of *Salmonella*-infected mice exhibited inflammatory cell infiltration, while miR-5112 agomiR administration dramatically reduced the accumulation of inflammatory cells compared with the administration of PBS (**Figure 5C**). These results indicate that miR-5112 agomiR-treated mice showed a reduced inflammatory response compared with PBS-treated mice during *Salmonella* infection. Furthermore, miR-5112 agomiR-treated mice had significantly reduced *Salmonella* loads in ileum and colon tissues compared with PBS-treated mice at 8 h post-infection (**Figure 6A**). After 4 days of *Salmonella* infection, the bacterial load in the ileum of miR-5112 agomiR-treated mice was still lower than that in PBS-treated mice (**Figure 6A**). Finally, the administration of the miR-5112 agomiR alleviated the loss of body weight (**Figure 6B**) and delayed the death of mice infected with *Salmonella* (**Figure 6C**).

### Exogenous miR-5112 AgomiR Alleviates Dextran Sulfate Sodium (DSS)-Induced Colitis

**Figure 7:**
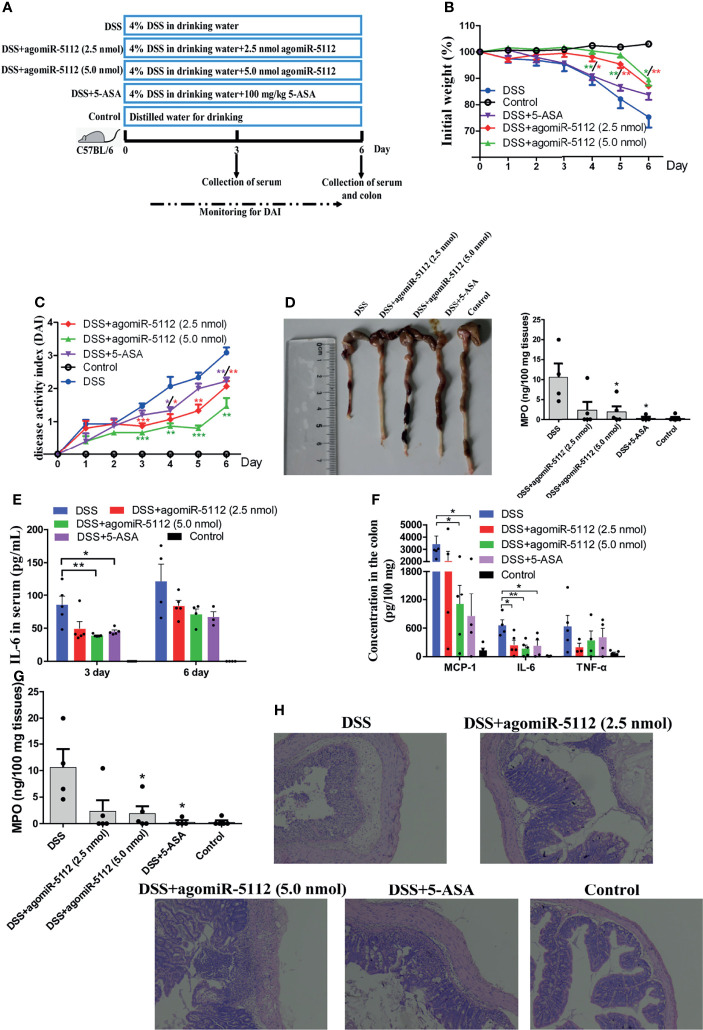
Exogenous miR-5112 agomiR alleviates DSS-induced colitis in mice. The colitis model was induced with 4% DSS in C57BL/6 mice (n=5/group). The therapeutic effect of agomiR-5112 on colitis was evaluated. 5-ASA was used as a positive control to treat the colitis. **(A)** Schematic diagram of the experimental design for 4% DSS-induced colitis and treatment. **(B)** Changes in the body weight of mice were recorded during DSS exposure. The weight changes were calculated as a percentage of the initial weight. **(C)** DAI was recorded to evaluate the severity of colitis during DSS exposure. DAI = (weight loss score + diarrhea score + bleeding score)/3. **(D)** The colon was photographed and its length was measured at 6 d after DSS induction. Cytokines in serum **(E)** and colon tissues **(F)** were determined using the BD™ CBA Mouse Inflammation Kit. **(G)** MPO levels in the colon were also determined using the Mouse MPO ELISA Kit according to the manufacturer’s instructions. **(H)** Histological analysis of the mouse colon at 6 d after DSS induction was examined by H&E staining. The results were observed at 100× magnification using an optical microscope. All data are expressed as the mean ± SEM. *P < 0.05; **P < 0.01; ***P < 0.001 versus DSS group.

DSS-induced colitis model is widely used because of its simplicity and many similarities with human ulcerative colitis (UC) which characterized by both acute and chronic inflammation of the intestine (26). miR-5112 in peritoneal macrophages and colon tissue was decreased after mice exposed to DSS for 6 days (**Figure S5**). We hypothesized that the administration of miR-5112 agomiR could also alleviate colitis. The ulcerative colitis model was developed by treating mice with 4% DSS. As shown in **Figure 7B**, mice in the DSS group exhibited significant body weight loss compared to the control group. The administration of miR-5112 agomiR (2.5 and 5.0 nmol) or 5-aminosalicylic acid (5-ASA, positive control) could significantly attenuate the body weight loss compared with the DSS group. The disease activity index (DAI) scores, an indicator of colitis, were significantly higher in the DSS group compared to the control group (**Figure 7C**). The administration of miR-5112 agomiR (2.5 and 5.0 nmol) or 5-ASA significantly reduced the DAI scores compared with the DSS group. In addition, DSS typically caused colonic shortening, while the administration of miR-5112 agomiR (2.5 and 5.0 nmol) or 5-ASA significantly reduced DSS-induced colon shortening (**Figure 7D**). Systemic and local inflammation was also evaluated by measuring the levels of cytokines and MPO in the serum and colon tissues. As shown in **Figure 7E**, the levels of IL-6 in serum were significantly increased in the DSS group at days 3 and 6 after DSS treatment. However, the increased IL-6 in serum levels could be suppressed by the administration of miR-5112 agomiR or 5-ASA. Additionally, the inflammatory cytokines IL-6, MCP-1, TNF-α, and MPO (an indicator of neutrophil influx and acute inflammation) induced by DSS in colon tissues were also reduced by miR-5112 agomiR or 5-ASA treatment (**Figures 7F, G**). We further performed histological analysis of the colon to assess the severity of colitis. Compared with the colon in the control group, the DSS group showed massive epithelial destruction, submucosal edema, and inflammatory cell infiltration in the submucosa and muscular layer (**Figure 7H**). In contrast, the administration of miR-5112 agomiR (2.5 and 5 nmol) or 5-ASA significantly alleviated these deteriorating pathological alterations (**Figure 7H**). These results demonstrated that miR-5112 agomiR could alleviate *Salmonella*-induced inflammation, as well as the microflora-induced chronic colitis.

## Discussion

miRNAs are small non-coding RNAs that regulate gene expression by targeting the 3’UTR mRNA. To date, more than 30, 000 miRNAs have been identified in at least 206 species (27). However, despite this, the biological functions of the majority of these miRNAs remain unknown. Inflammation can be induced by the recognition of foreign pathogens *via* PRRs, including TLRs, resulting in the release of downstream proinflammatory cytokines and chemokines. It acts as a first line of defense against infection (28) and needs to be tightly regulated. In this study, we identified miR-5112 as a novel regulator of the flagellin-triggered inflammatory response. First, we found that flagellin stimulation induced miR-5112 downregulation in murine BMDCs and spleen DCs (both *in vitro* and *in vivo*). Second, we confirmed that enforced miR-5112 expression could decrease inflammatory cytokine production, accompanied by a reduction of IKKγ in BMDCs induced by flagellin. Third, we demonstrated that IKKγis the miR-5112 target in the TLR5 signaling pathway. Therefore, we present a potential regulatory mechanism for the DC activation induced by flagellin (**Figure 4D**).

TLR5, a member of the TLR family, is responsible for the recognition of bacterial flagellin, resulting in the production of proinflammatory cytokines and the upregulation of costimulatory molecules in immune cells (2). Our data showed that BMDCs isolated from C3H/HeJ mice produced more IL-12 p40 and upregulated surface markers (**Figure S2**) following flagellin treatment, consistent with previous reports (29). These data indicate that BMDCs from C3H/HeJ mice were activated by flagellin. However, Means et al. found that BMDCs isolated from C57BL6 mice were less responsive to flagellin (30). The varying TLR5 expression levels in BMDCs from different mice may result in a differential response to flagellin in murine BMDCs. TLR5 transcripts were detected in BMDCs derived from C3H/HeJ mice rather than in C57BL6 mice (31). Therefore, BMDCs derived from C3H/HeJ mice were used as a model to study the regulatory mechanism responsible for murine BMDC activation induced by flagellin.

Accumulating evidence has shown that miRNAs are involved in regulating DC function. miR-155 has been shown to be upregulated in DCs upon LPS stimulation and to limit the over-production of inflammatory cytokines by targeting TAB2 (32). miR-146a can also regulate inflammatory cytokine responses during DC activation by TLR recognition (33, 34). In our study, miR-5112 was shown to be downregulated after flagellin stimulation in BMDCs and spleen DCs both *in vitro* and *in vivo*. This finding indicates that miR-5112 may participate in the regulation of DC activation induced by flagellin. The exact mechanism of miR-5112 reduction after flagellin stimulation requires further investigation.

miR-5112 was initially identified in B cells by deep sequencing (35). However, its function remains unknown. Our results showed that the overexpression of miR-5112 inhibited IL-12 p40 production by BMDCs induced by flagellin (**Figure 2A**), suggesting that miR-5112 could function as a negative regulator of IL-12 p40 production in BMDCs. We also showed that IKKγ was a direct target of miR-5112 and could be negatively regulated at the post-transcriptional level by miR-5112. IKKγ is the regulatory subunit of the inhibitor of the IκB kinase (IKK) complex, which activates NF-κB, resulting in the release of inflammatory cytokines (36). The inhibition of IKKγ can block TLR-dependent NF-κB activation, resulting in a reduction in inflammatory cytokines (37). Similar to our study, Unlu et al. demonstrated that IKKγ can be targeted by another miRNA, miR-34c, to regulate the NF-κB inflammatory pathway in human peripheral blood mononuclear cells (38). However, it is also possible that some other potential miR-5112 targets also modulate the flagellin-triggered inflammatory cytokines in DCs.

Flagellin, recognized by TLR5, can induce inflammatory cytokine production and initiate the innate immune response. However, the over-production of inflammatory cytokines can induce injury and pathological lesions. The intravenous administration of flagellin in mice can induce severe acute lung inflammation (39). High-dose flagellin can cause acute inflammatory responses and induce liver injury in mice (40). Our data showed that the overexpression of miR-5112 could significantly decrease the inflammation caused by flagellin-induced cytokine production (**Figure 3**), indicating that miR-5112 may be utilized as a potential therapeutic tool to control inflammatory diseases.

Recently, evidence has increasingly suggested that miRNAs play an important role in the host response to bacterial infection (41–43). Let-7 can be downregulated to induce the release of cytokines IL-6 and IL-10 to participate in the regulation of the immune response to *Salmonella* infection in macrophages (44). The inhibition of miR-29 activity in mice can enhance the expression of IFN-γ and decrease the *L. monocytogenes* burden in infected mice (45). The enforced expression of miR-302b can inhibit inflammatory cytokine production and airway leukocyte infiltration, thereby alleviating lung injury and increasing survival in *P. aeruginosa*-infected mice (46). In our study, we also found that miRNAs can regulate the host response to bacterial infection. The enforced expression of miR-5112 decreased inflammatory cytokine production in serum and alleviated inflammatory cell infiltration in the liver and cecum during *Salmonella* infection (**Figure 5**). In addition, we also found that enforced miR-5112 expression could reduce the *Salmonella* burden and delay death in *Salmonella-*infected mice (**Figure 6**). These results indicate that miR-5112 plays an important role in the host response to defence against *Salmonella* infection.

DSS-induced colitis model is widely used in human ulcerative colitis (UC) to decipher underlying mechanisms of pathogenesis as well as to evaluate number potential therapeutics (26). Ulcerative colitis, an inflammatory bowel disease (IBD), is a chronic inflammatory disease of the gastrointestinal tract characterized by intestinal epithelial inflammation and injury (47). UC is an important public health problem, and the number of patients is currently increasing in developed countries. Although the cause of IBD is not fully known, several lines of evidence suggest that the NF-κB pathway plays a critical role in the pathogenesis of UC (48). It has been reported that the mechanism of conventional therapeutic agents for UC, such as 5-aminosalicylic acid (5-ASA), involves the inhibition of the NF-κB signalling pathway (49). miRNA-mediated gene regulation has been observed in various UC processes. miR-10a has been demonstrated to regulate chronic intestinal inflammation by inhibiting the expression of IL-12/IL-23p40 and NOD2, as well as Th1 and Th17 cell functions in IBD (50). Dai et al. found that the intracolonic delivery of miR-193a-3p can significantly ameliorate DSS-induced colitis by suppressing the NF-κB pathway (51). miR-146b also improves intestinal injury in DSS-induced colitis by regulating the NF-κB pathway (20). In our study, we evaluated the effect of miR-5112, which could target IKKγ to regulate the NF-κB signalling pathway in DSS-induced colitis in C57BL/6 mice. We found that the enforced expression of miR-5112 could significantly improve the clinical symptoms of DSS-induced colitis, including body weight loss, colon length shortening, disease activity index increase, and histological lesions (**Figure 7**). In addition, miR-5112 overexpression also had a remarkable effect on decreasing DSS-induced secretion of proinflammatory cytokines both in serum and colon tissue and MPO (an indicator of neutrophil influx and acute inflammation) in the colon (**Figure 7**). The effect of miR-5112 was comparable to that of the positive control 5-ASA, which has been used for more than 30 years in the treatment of inflammatory bowel disease. These results suggest that miR-5112 could be used as an alternative therapeutic tool for the treatment of DSS-induced colitis model.

In conclusion, our findings suggest a potential regulatory mechanism for inflammatory production during flagellin-induced DC activation (**Figure 4D**). We demonstrated that miR-5112 expression was downregulated in DCs after flagellin stimulation, which in turn promoted the production of inflammatory cytokines by targeting IKKγ. These findings provide useful insights into the molecular mechanisms underlying flagellin-triggered inflammatory responses. Furthermore, we demonstrated that the administration of miR-5112 agomiR could also decrease the inflammatory response induced by flagellin or *Salmonella* in mice. Finally, miR-5112 could also reduce DSS-induced secretion of proinflammatory cytokines and improve the clinical signs of DSS-induced colitis, indicating that miR-5112 represents a potential alternative therapeutic tool for bacterial infections and inflammatory bowel diseases.

## Data Availability Statement

The datasets presented in this study can be found in online repositories. The names of the repository/repositories and accession number(s) can be found below: https://www.ncbi.nlm.nih.gov/geo; GSE188656.

## Ethics Statement

The animal study was reviewed and approved by Animal Welfare and Ethics Committees of Yangzhou University.

## Author Contributions

ZP and XJ conceived and designed research. XK, YJ, and YZ performed all experiments. XK, CM, LS, and ZP analyzed the data. XK wrote the manuscript. XZ participated in the design of the study and review of manuscript. All authors read and approved the final manuscript.

## Funding

This work was supported by the National Natural Science Foundation of China (31902278, 31972685, and 31172299), the China Postdoctoral Science Foundation (2018M642333), the Priority Academic Program Development of Jiangsu Higher Education Institutions (PAPD), and the Graduate Student Research and Innovation Project of Jiangsu Province (KYLX15_1381).

## Conflict of Interest

The authors declare that the research was conducted in the absence of any commercial or financial relationships that could be construed as a potential conflict of interest.

## Publisher’s Note

All claims expressed in this article are solely those of the authors and do not necessarily represent those of their affiliated organizations, or those of the publisher, the editors and the reviewers. Any product that may be evaluated in this article, or claim that may be made by its manufacturer, is not guaranteed or endorsed by the publisher.

## Supplementary Material

Supplementary Table S1 Primers used for qRT-PCR analysis of miRNAs.

Supplementary Figure S1 Purity analysis of BMDCs by FCM. BMDCs generated from C3H/HeJ murine bone marrow progenitors were cultured in the presence of rmGM-CSF and rmIL-4. Next, the cells were harvested and sorted with anti-CD11c-coated magnetic beads using an auto-MACS system. The purity of the CD11c^+^ BMDCs was analyzed using FCM by staining with PE-labeled CD11c. **(A)** BMDCs before sorting, **(B)** sorted BMDCs.

Supplementary Figure S2 Activation of BMDCs induced by flagellin. The sorted BMDCs were cultured with 100 ng/ml flagellin or medium alone for 24 h. The expression of surface markers (CD40, CD80, and CD86) in BMDCs was analyzed by FCM. FCM histograms indicate the fluorescence intensity of surface marker **(A)**. The expression of surface markers is also presented as mean fluorescence intensity (MFI) **(B)**. IL-12 p40 production in culture supernatants of BMDCs was measured by ELISA using a mouse IL-12 p40 ELISA Kit **(C)**. The data shown represent the means ± SEM from 3 independent experiments. **P* < 0.05, ****P* < 0.001.

Supplementary Figure S3 The analysis of miR-5112 expression in peritoneal macrophages after the mice were infected with *S. enteritidis*. The mice were administered to 7.5 mg of streptomycin by oral gavage. After 24 h, the mice were infected with 5 ×10^4^ CFU of C50336 (100 -μl of bacterial suspension in PBS) or treated with 100 μl of sterile PBS (control) by oral gavage. At 8 hours after *Salmonella* infection, three mice per group were sacrificed, and the peritoneal macrophages were collected. The expression of miR-5112 in peritoneal macrophages were determined by qRT-PCR.

Supplementary Figure S4 The analysis of miR-5112 level in organs of mice after injection with agomiR-5112. The mice were intraperitoneally injected 2.5 nmol of agomiR-5112 on three consecutive days. Then, three mice per group were sacrificed, and the peritoneal macrophages, spleen and colon were collected. The miR-5112 levels in organs were determined by qRT-PCR.

Supplementary Figure S5 The analysis of miR-5112 expression in peritoneal macrophages and colon of DSS-induced colitis mice. The colitis model was induced with 4% DSS in C57BL/6 mice. The mice (n=3/group) were sacrificed, and the peritoneal macrophages, and colon were collected after mice exposed to DSS for 6 days. The miR-5112 levels in organs were determined by qRT-PCR.
