# Supplementary material for: MicroRNA-5112 Targets IKKγ to Dampen the Inflammatory Response and Improve Clinical Symptoms in Both Bacterial Infection and DSS-Induced Colitis
Source: Front Immunol. 2022 Feb 10;13:779770. doi: 10.3389/fimmu.2022.779770 (PMC8866336; doi:10.3389/fimmu.2022.779770)
Supplement: Supplementary Table S1 — Primers used for qRT-PCR analysis of miRNAs. [file Table_1.docx]

| miRNAs | Primers | Primer sequences (5’-3’) | Usage |
| --- | --- | --- | --- |
| miR-5112 | miR-5112-RT | GTCGTATCCAGTGCAGGGTCCGAGGTATTCGCACTGGATACGACGTGCTC | reverse transcription |
|  | miR-5112-F | ATAGCTCAGCGGGAGAGCA | qPCR |
|  | miR-5112-R | GTGCAGGGTCCGAGGT | qPCR |
| miR-193-5p | miR-193-5p-RT | GTCGTATCCAGTGCAGGGTCCGAGGTATTCGCACTGGATACGACTCATC | reverse transcription |
|  | miR-193-5p-F | TGGGTCTTTGCGGGCAA | qPCR |
|  | miR-193-5p-R | GTGCAGGGTCCGAGGT | qPCR |
| miR-466i-3p | miR-466i-3p-RT | GTCGTATCCAGTGCAGGGTCCGAGGTATTCGCACTGGATACGACTAGTG | reverse transcription |
|  | miR-466i-3p-F | GCCATACACACACACATACACAC | qPCR |
|  | miR-466i-3p-R | GTGCAGGGTCCGAGGT | qPCR |
| miR-3091-5p | miR-3091-5p-RT | GTCGTATCCAGTGCAGGGTCCGAGGTATTCGCACTGGATACGACGCGGGC | reverse transcription |
|  | miR-3091-5p-F | ATAGCTCAGCGGGAGAGCA | qPCR |
|  | miR-3091-5p-R | GTGCAGGGTCCGAGGT | qPCR |
| U6 | U6-RT | AACGCTTCACGA ATTTGCGT | reverse transcription |
|  | U6-F | CTCGCTTCGGCAGCACA | qPCR |
|  | U6-R | AACGCTTCACGA ATTTGCGT | qPCR |

Table S1. Primers used for qRT-PCR analysis of miRNAs
