# Supplementary figures and images for: MicroRNA-5112 Targets IKKγ to Dampen the Inflammatory Response and Improve Clinical Symptoms in Both Bacterial Infection and DSS-Induced Colitis
Source: Front Immunol. 2022 Feb 10;13:779770. doi: 10.3389/fimmu.2022.779770 (PMC8866336; doi:10.3389/fimmu.2022.779770)

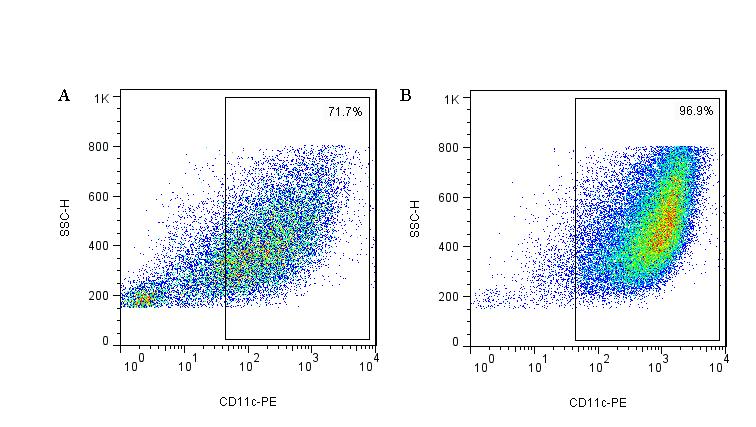

Supplement: Supplementary Figure S1 — Purity analysis of BMDCs by FCM. BMDCs generated from C3H/HeJ murine bone marrow progenitors were cultured in the presence of rmGM-CSF and rmIL-4. Next, the cells were harvested and sorted with anti-CD11c-coated magnetic beads using an auto-MACS system. The purity of the CD11c+ BMDCs was analyzed using FCM by staining with PE-labeled CD11c. (A) BMDCs before sorting, (B) sorted BMDCs. [file Image_1.tif]

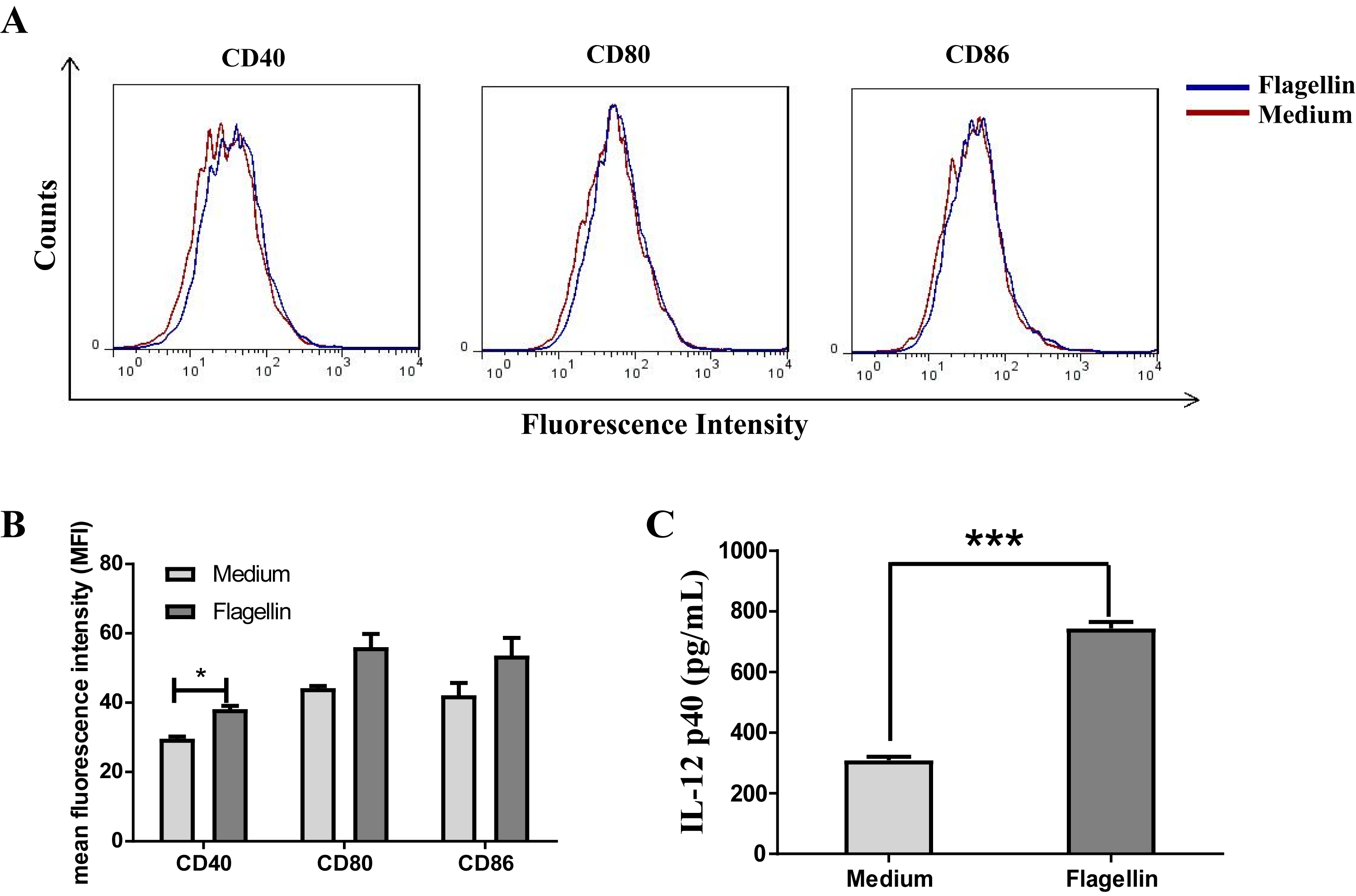

Supplement: Supplementary Figure S2 — Activation of BMDCs induced by flagellin. The sorted BMDCs were cultured with 100 ng/ml flagellin or medium alone for 24 h. The expression of surface markers (CD40, CD80, and CD86) in BMDCs was analyzed by FCM. FCM histograms indicate the fluorescence intensity of surface marker (A). The expression of surface markers is also presented as mean fluorescence intensity (MFI) (B). IL-12 p40 production in culture supernatants of BMDCs was measured by ELISA using a mouse IL-12 p40 ELISA Kit (C). The data shown represent the means ± SEM from 3 independent experiments. *P < 0.05, ***P < 0.001. [file Image_2.tif]

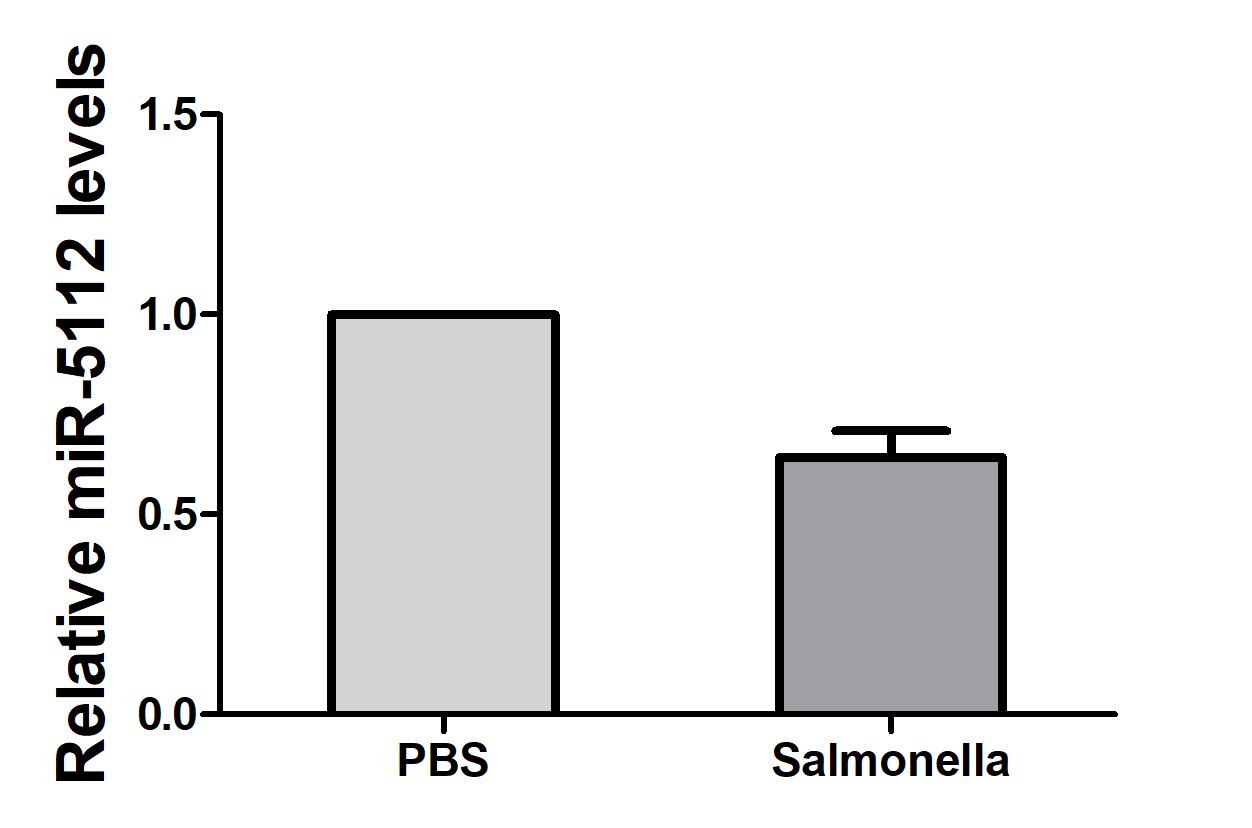

Supplement: Supplementary Figure S3 — The analysis of miR-5112 expression in peritoneal macrophages after the mice were infected with S. enteritidis. The mice were administered to 7.5 mg of streptomycin by oral gavage. After 24 h, the mice were infected with 5 ×10^4^ CFU of C50336 (100 -μl of bacterial suspension in PBS) or treated with 100 μl of sterile PBS (control) by oral gavage. At 8 hours after Salmonella infection, three mice per group were sacrificed, and the peritoneal macrophages were collected. The expression of miR-5112 in peritoneal macrophages were determined by qRT-PCR. [file Image_3.jpeg]

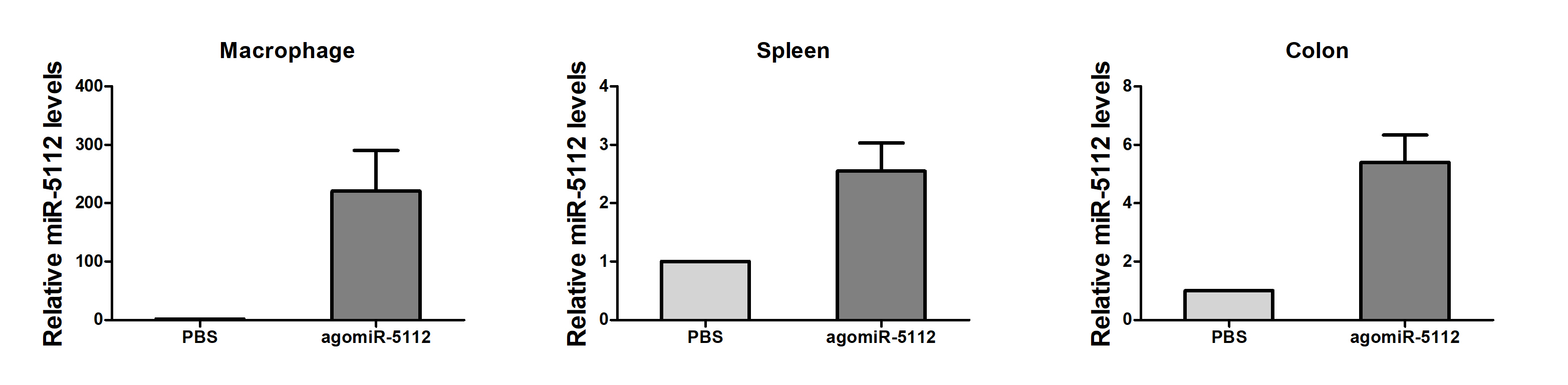

Supplement: Supplementary Figure S4 — The analysis of miR-5112 level in organs of mice after injection with agomiR-5112. The mice were intraperitoneally injected 2.5 nmol of agomiR-5112 on three consecutive days. Then, three mice per group were sacrificed, and the peritoneal macrophages, spleen and colon were collected. The miR-5112 levels in organs were determined by qRT-PCR. [file Image_4.tif]

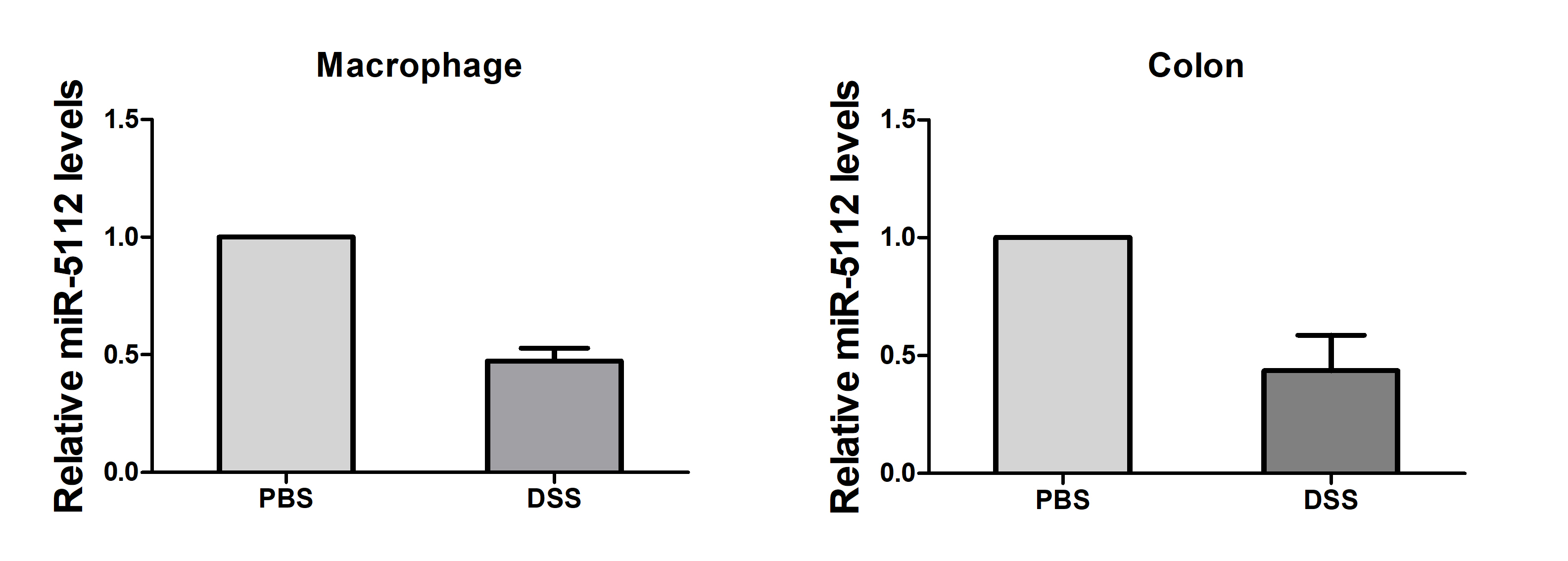

Supplement: Supplementary Figure S5 — The analysis of miR-5112 expression in peritoneal macrophages and colon of DSS-induced colitis mice. The colitis model was induced with 4% DSS in C57BL/6 mice. The mice (n=3/group) were sacrificed, and the peritoneal macrophages, and colon were collected after mice exposed to DSS for 6 days. The miR-5112 levels in organs were determined by qRT-PCR. [file Image_5.jpeg]
